# Long-term follow-up of disability pensioners having musculoskeletal disorders

**DOI:** 10.1186/1471-2458-9-407

**Published:** 2009-11-10

**Authors:** Liv H Magnussen, Liv I Strand, Jan S Skouen, Hege R Eriksen

**Affiliations:** 1 Unifob Health, Bergen, Norway; 2 Department of Public Health and Primary Health Care, University of Bergen, Bergen, Norway; 3 The Multidisciplinary Outpatient Spine Clinic, Haukeland University Hospital, Bergen, Norway; 4 Hemil, Research Centre for Health Promotion, University of Bergen, Bergen, Norway

## Abstract

**Background:**

Previously we have conducted a randomised controlled trial (RCT) to evaluate the effect of a brief cognitive behavioural program with a vocational approach aiming to return disability pensioners with back pain to work, as compared to no intervention. One year after the intervention, 10 participants (22%) who received the program and 5 (11%) in the control group reported to have entered a return to work process. The aims of this study were to evaluate long-term effects of the intervention, and compare this effect to 2 reference populations not participating in the original trial.

**Methods:**

Three groups of disability pensioners were investigated: 1) Disability pensioners having back pain (n = 89) previously participating in the RCT (randomized to either a brief cognitive behavioural intervention or to a control group), 2) 342 disability pensioners having back pain, but refusing to participate in the study and 3) 449 disability pensioners having other musculoskeletal disorders than back pain. Primary outcome was return to work, defined as a reduction in payment of disability pension.

**Results:**

Only 2 of 89 (2.3%) participants from the RCT had reduced disability pension at 3-years follow-up, both from the control group. None of the participants that had been in a process of returning to work after 1 year had actually gained employment at 3-years follow-up. In the 2 groups not participating in the previous RCT, only 4 (1.2%) and 8 (1.6%) had returned to work after 3 years respectively.

**Conclusion:**

The number of pensioners who returned to work was negligible in all groups regardless of having participated in a cognitive behavioural intervention or not.

## Background

The large number of individuals leaving work prematurely due to ill health is alarming. In Norway, the number of disability pensioners increased by 26% from 1996 to 2003 [1]. The figures are still rising and amounted to about 11% of the adult population in 2008 [2]. The major causes of work disability in Norway are musculoskeletal and mental disorders. This study is a long-term follow-up of disability pensioners having musculoskeletal disorders regarding to which extent they eventually return to work.

The time from leaving work and until disability pension (DP) is granted will in Norway take at least 3 years [2]. However, a considerable proportion of individuals ending up with a DP have experienced a much longer disability history moving in and out of different insurance benefits for years. Approximately 45% of all persons granted DP receive some kind of insurance or social benefit for as long as 10 years before eventually being granted a permanent DP [3], underscoring the prolonged illness of the study population.

DP is a permanent allowance in Norway. However, the pensioners are allowed to earn a basic income of about 8100 EUR per year without cuts in pension [4]. If this limit is exceeded, the pension will be reduced correspondingly. To improve the likelihood of returning to work, economic incentives for the employer and employee have been introduced, including arrangements stimulating to the combination of work and DP. In 2003, the National Insurance Administration (NIA) interviewed 23000 disability pensioners about the prospect of re-entering work [5]. Of these, 10300 (46%) expressed a motivation for trying. However, no actual re-employment was observed after 3 years, and the authors concluded that the observation period had been too short. Prolonged sick leave is known to reduce the chance of ever returning to work [6,7], and after becoming a disability pensioner, future work perspectives seems even more pessimistic. According to OECD, less than 1% of the disability pensioners re-enter work each year [8], and studies evaluating the effect of vocational rehabilitation programs among disability pensioners are scarce. Motivation for work [9-11], improved health and self-esteem, and close support have been shown to increase the likelihood of re-entering work-related activity [10,12]. Other determinants are regulations concerning disability compensation, insurance policies, employer obligations and governmental programs [8,13,14].

We have recently conducted a randomized controlled trial (RCT) to evaluate the effect of a brief cognitive behavioural program with a vocational approach aiming to motivate individuals receiving DP due to back pain to re-enter work [11]. The study was carried out as a group intervention combining lectures and motivational interviewing. Counsellors from the social insurance office and work office provided information and outlined options for combining health-adjusted work and disability benefit, and a medical examination and follow-up were offered by physicians. The effect of the intervention was not statistically significant regarding a return to work, but still, twice as many in the intervention group (n = 10) reported to have entered a work-related process compared to the controls (n = 5). We wanted to investigate closer whether this positive trend would continue or increase over time, as it may take more than 1 year for the pensioners to reach permanent employment.

The 87 participants in our previous RCT was a highly selected sample (only 20% of the invited population). We were concerned that our sample was more or less motivated to return to work compared to the population they were recruited from, and had the opportunity to investigate whether our sample differed from this population regarding a return to work. As there is a substantial overlap between LBP diagnoses and other diagnoses related to musculoskeletal pain [15,16] we also included another group of disability pensioners with musculoskeletal pain, to investigate whether the pensioners in this group were more or less likely to return to work than the original study group of back pain pensioners.

The primary aim of the present study was to investigate whether disability pensioners reporting to have entered a return to work process at 1-year follow-up [11] actually went back to work within the next 2 years. The secondary aim was to investigate if there were differences in return to work between the participants in the RCT and the population they were recruited from, and the third aim was to examine if disability pensioners with a back pain diagnosis differed from pensioners with other musculoskeletal disorders.

## Methods

### Design and description of the study groups

Three groups of disability pensioners were investigated. 1) Disability pensioners having back pain (n = 89) previously participating in the RCT (randomized to either a brief cognitive behavioural intervention (n = 45) or to a control group (n = 44)) [11], 2) 342 disability pensioners with back pain who were invited, but not willing to participate in the previous RCT and 3) 449 disability pensioners with other musculoskeletal disorders than back pain, not invited to the above mentioned study. Mean duration of DP varied from 9.8 to 11.6 years in the groups.

Inclusion criteria were age under 55 years, receivers of DP for at least 1 year due to back pain or another musculoskeletal disorder. Register data including age, gender, duration of DP and current DP status 1 and 3 years after the intervention was available.

**Figure 1:**
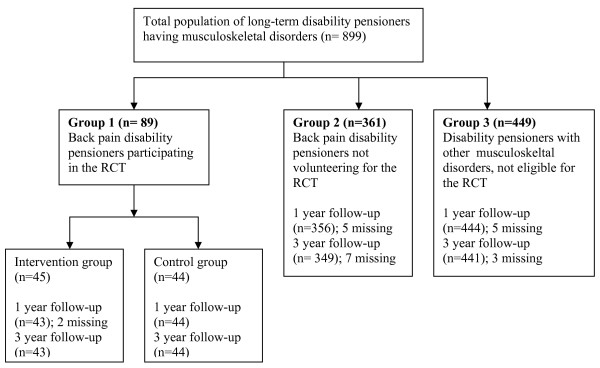
**Long-term follow-up of disability pensioners with musculoskeletal disorders**.

The eligible population of disability pensioners was recruited from the National Insurance Administration (NIA). Recruitment and follow-up of the study groups are shown in Figure 1.

### Ethics

The study was approved by the Norwegian Ethics Committee for Medical Research, Health Region West, and performed according to the Helsinki Declaration.

### Outcome

The main outcome was return to work defined as reduced payment of DP measured by register data from the NIA. Reductions in DP payment will in Norway only take place when the pensioner increases his income.

### Statistical analysis

SPSS version 15.0 was used for the statistical analysis. Descriptive statistics were used to describe socio-demographic and insurance data (demographic information, number of years receiving DP, benefit rates) of the study groups. Analysis of variance and Tukey HSD Post Hoc Test was used to examine differences in socio-demographic data between the respective groups. Differences in payment of DP were counted and compared manually due to the low numbers.

## Results

Only 14 out of 899 disability pensioners (1.6%) were registered by reductions in payment of DP at 3-years follow-up.

### Participants in the RCT at 3-year follow-up (Group 1)

At 3-year follow-up none of the participants in the intervention group had returned to work. Hence, the 15 participants reporting to have entered a return to work process after 1 year had not succeeded in getting employed after 3 years. Two participants in the control group had a reduction in DP at 3-years follow-up. One had 50% reduction, and the other 20% reduction (Table 1).

**Table 1:** Characteristics of the study samples based on register data

**Variables**	**Group 1** **Participants RCT** **(n = 89)**		**Group 2** **Non-participants** **(n = 361)**	**Group 3** **Musculoskeletal** **(n = 449)**
			
	**Intervention** **(n = 45)**	**Control** **(n = 44)**		
Age, yrs: mean (SD)	49.1 (6.4)	49.0 (4.5)	50.0 (5.7)	49.6 (5.9)
Gender, female: n (%)	26 (60.0)	30 (69.8)	200 (55)	355 (79)
DP, yrs: mean (SD)	9.8 (4.8)	11.6 (5.8)	11.0 (5.9)	9.8 (4.8)
DP at 1-year follow-up*				
Full time employed: n (%)	-	-	-	-
0<DP≤50: n (%)	-	-	-	1 (0.2)
50<DP<100: n (%)	1 (2.3)	2 (4.6)	4 (1.2)	2 (0.4)
Full time DP	42 (97.7)	42 (95.5)	352 (97.5)	441 (98.2)
DP at 3-year follow-up*				
Full time employed: n (%)	-	-	-	1 (0.2)
0<DP≤50: n (%)	-	1 (2.3)	-	-
50<DP<100: n (%)	-	1 (2.3)	4 (1.2)	8 (1.6)
Full time DP	43 (100)	42 (95.5)	345 (98.8)	432 (98.4)

Group 1: Disability pensioners with back pain participating in the RCT, Group 2: Disability pensioners with back pain, invited, but not willing to participate in the RCT, Group 3 Disability pensioners with other musculoskeletal disorders not invited to participate in the RCT. DP = disability pension. *Numbers are given in valid percent. Two were missing in the intervention group, 12 were missing in Group 2 and 8 were missing in Group 3 at 3 year follow-up.

### Disability pensioners with back pain and musculoskeletal disorders not participating in the RCT at 3-years follow up (Groups 2 and 3)

Four (1.2%) of the non-participants with back pain were registered with only minor reductions in payment of DP after 3 years. In the musculoskeletal group, only 1 pensioner (0.2%) had fully returned to work (100% reduction in DP) and 8 pensioners (1.6%) had less than 50% reduction in payment of DP at 3-years follow-up (Table 1).

### Differences between the 3 groups

The RCT participants (n = 89) did not differ from the non-participants (n = 342) or the musculoskeletal group (n = 449) regarding age (F (2,895) = 1.2, p = 0.3) (Table 1). In the musculoskeletal group, 79% were women compared to 65% in the group of RCT participants (60% in the intervention group and 69.8% in the control group) and 55% in non-participants (F (2,896) = 27.5, p < 0.01). There was also a small difference between the groups in number of years receiving DP (F (2, 894) = 4.9, p = 0.007); RCT participants had received DP for an average of 10.6 years (SD 4.9), while corresponding numbers in non-participants and the musculoskeletal group were 11.0 (SD 5.9) and 9.8 years (SD 4.8), respectively.

## Discussion

This study confirms that it is a great challenge to re-enter work after becoming a disability pensioner. Of 899 disability pensioners with musculoskeletal disorders, only 1 single person had returned to full time work, while 14 had small reductions in payment of DP at 3-year follow-up. This result also included a group of disability pensioners who had participated in a previous cognitive behavioural program with a vocational approach. Despite a positive trend regarding progress towards work after 1 year, none of these individuals actually returned to work within the next 2 years. The poor results are in line with other reports on re-employment efforts among disability pensioners [8,17].

Despite no effect in the main outcome, the initial conclusion after 1-year follow-up suggested a modest success as several participants reported to have entered into a work-related process. We expected that a proportion of the participants who had been in such a process would actually have returned to work at the 3-years follow-up. This assumption is supported by findings in other studies showing a moderate effect of brief interventions in terms of returning sick listed persons with back pain to work [18,19]. However, no previous studies have examined long-term effects of intervention programmes directed towards disability pensioners. Therefore, the present study was carried out to examine whether an intervention would bring on a more permanent or even increased effect in the long run. The negative result of the 3-years follow-up was therefore an important, but rather disappointing finding.

Work-related interventions for this chronic group should maybe have been more extensive and individually adjusted than the brief intervention we offered. A strategy based on a closer collaboration with possible work places, and a more comprehensive program in general, including more extensive support from all parts involved in the process, might have increased the success rate. Another important factor for success in this field is the individual's positive beliefs and expectancy regarding recovery and employment [20-22]. Previously, we have reported that a majority of the disability pensioners had negative beliefs regarding returning to work [23]. In a qualitative study of the same population we found indications that negative beliefs were linked to earlier negative experiences with the workplace. These experiences included lack of willingness from management and colleagues to make work adjustments [10]. In addition, uncertainties about future health, financial consequences and work skills have also been reported as barriers against returning to work [10,24]. Identifying these barriers and directly addressing them in the intervention programmes, may increase the likelihood of re-entering work [25].

Pensioners being in a work related process might have experienced only a small, if any, increase in income, and this fact has been described as de-motivating [10]. A more substantial increase in income may result in a better motivation and more lasting effects. In Norway, disability pensioners are allowed to earn a limited income without any reduction in DP, and some will cope well within this frame of activity. The present study was solely based on insurance data, and therefore we do not know if any pensioners had obtained this additional income. As Norway has a very generous DP [8], our results might not be comparable to other countries where being employed yields a larger economic benefit.

Failure of lasting results might also be due to the insurance policy. An initial attempt to try out for work will often be made with economic support from the social insurance offices. Transformation to a permanent job paid for by an employer has been shown to be difficult, as employers prefer to hire employees without a history of sick leave and disability if given the option.

We found no difference in long time re-employment between different diagnostic groups or between intervention and control groups. Neither age, gender, time upon DP, nor being submitted to intervention or not, seemed to influence the negative result. We found a difference between the groups regarding duration of DP varying from 9.8 to 11 years, but this small difference has probably no practical impact. We regard the long time span of disability in itself as the main obstacle to a successful return to work, not the diagnosis or vocational interventions provided. It is tempting to speculate whether the pensioners concluded that they were comfortable with their life situation as it was. Most have many health complaints [23] and may consider working life as too demanding, and was therefore, after several years, have come to acceptance with their situation. In a previous study, pensioners expressed that they appreciated the safety of the DP, and were reasonably satisfied with their life situation [10].

### Methodological considerations

We used reduction in payment of DP as an indicator of having returned to work. If disability pensioners exceed the limit of income he or she is allowed to earn, the pension will be reduced correspondingly. In Norway will be no other reason for the pension to be reduced or removed. Thus, we could assume that a reduction in DP payment meant that the pensioner earned more than the allowed amount. A possible limitation in our study was that we did not have access to more detailed information about the pensioners. We do not know whether the 3 study groups were comparable regarding factors like expectancy, level of education and previous occupation. Also, considering the small number of participants who had entered a return to work process after 1 year, we find that a qualitative study outlining the pensioners' experiences of the unsuccessful return to work process could have yielded additional important information to the outcome.

## Conclusion

The results of our study showed that return to work after 3 years in a chronic group of disability pensioners was negligible, whether they had received a brief intervention or not. The modest, but promising trend at 1-year follow-up did not yield a lasting effect. These disappointing findings add to the impression that returning long-term disability pensioners to work is a very challenging task. Prevention or more comprehensive interventions at much earlier stages in the disability process seem necessary to prevent long-term DP in those who have a potential for a return to work.

## Competing interests

The authors declare that they have no competing interests.

## Authors' contributions

LHM contributed to conception and design of the study, data interpretation, and drafted the manuscript. LIS and JSS conceived of the study, participated in its design, and revised various versions of the manuscript. HRE conceived of the study and made substantial contribution to the conception and design of the study, the interpretation of data, and revised various versions of the manuscript. All authors read and approved the final manuscript.

## Pre-publication history

The pre-publication history for this paper can be accessed here:


